# Birthweight, childhood growth and risk of breast cancer in a British cohort

**DOI:** 10.1054/bjoc.2000.1370

**Published:** 2000-09-04

**Authors:** B L De Stavola, R Hardy, D Kuh, I dos Santos Silva, M Wadsworth, A J Swerdlow

**Affiliations:** Cancer and Public Health Unit, Department of Epidemiology and Population Health, London School of Hygiene and Tropical Medicine, Keppel Street, London, WC1E 7HT; MRC National Survey of Health and Development, Department of Epidemiology and Public Health, University College London, 1–19 Torrington Place, London, WC1E 6BT, UK

**Keywords:** birthweight, childhood growth, breast cancer, cohort study

## Abstract

We have examined the relationship between birthweight and risk of breast cancer, taking into account growth in childhood, using data on a total of 2221 women born in 1946 and followed up to 1997. Thirty-seven breast cancers occurred during follow-up. There was evidence of greater risk of breast cancer with greater birthweight (rate ratio = 1.76 (95% CI: 0.92, 3.35) for birthweight ≥ 3.5 kg vs birthweight < 3.5 kg), which was more marked at pre-menopausal ages (RR = 2.31, 95% CI: 0.93, 5.74). The relation with birthweight was not substantially confounded by any of the measured adult risk factors. A significant interaction was observed between the effects of birthweight and height at age 7 years. Relative to those born lighter than 3.5 kg, women who were heavy at birth (≥ 3.5 kg) and short or average at 7 years (< 1.22 m) had a 21% increase in breast cancer rates (RR = 1.21; 95% CI = 0.49–2.99), while women who were heavy at birth (≥ 3.5 kg) but tall at 7 years (≥ 1.22 m) had a four-fold increase (RR = 4.01; 95% CI = 1.82–8.83). These results suggest that the effect of birthweight on breast cancer risk may be modulated by childhood growth. © 2000 Cancer Research Campaign


*British Journal of Cancer* (2000) **83**(7), 964–968
© 2000 Cancer Research Campaign
doi: 10.1054/ bjoc.2000.1370, available online at http://www.idealibrary.com on IDEAL®

### Birthweight, childhood growth and risk of breast cancer in a British cohort

**BL De Stavola[1], R Hardy[2], D Kuh[2], I dos Santos Silva[1], M Wadsworth[2] and AJ Swerdlow[1]**

[1]Cancer and Public Health Unit, Department of Epidemiology and Population Health, London School of Hygiene and Tropical Medicine, Keppel Street, London WC1E 7HT; [2]MRC National Survey of Health and Development, Department of Epidemiology and Public Health, University College London, 1–19 Torrington Place, London WC1E 6BT, UK

**Summary** We have examined the relationship between birthweight and risk of breast cancer, taking into account growth in childhood, using data on a total of 2221 women born in 1946 and followed up to 1997. Thirty-seven breast cancers occurred during follow-up. There was evidence of greater risk of breast cancer with greater birthweight (rate ratio = 1.76 (95% CI: 0.92, 3.35) for birthweight ≥ 3.5 kg vs birthweight < 3.5 kg), which was more marked at pre-menopausal ages (RR = 2.31, 95% CI: 0.93, 5.74). The relation with birthweight was not substantially confounded by any of the measured adult risk factors. A significant interaction was observed between the effects of birthweight and height at age 7 years. Relative to those born lighter than 3.5 kg, women who were heavy at birth (≥ 3.5 kg) and short or average at 7 years (< 1.22 m) had a 21% increase in breast cancer rates (RR = 1.21; 95% CI = 0.49–2.99), while women who were heavy at birth (≥ 3.5 kg) but tall at 7 years (≥ 1.22 m) had a four-fold increase (RR = 4.01; 95% CI = 1.82–8.83). These results suggest that the effect of birthweight on breast cancer risk may be modulated by childhood growth. © 2000 Cancer Research Campaign

**Keywords**: birthweight; childhood growth; breast cancer; cohort study

There has been recent interest in the possibility that breast cancer can have a prenatal origin (Trichopoulos, 1990). Various studies have reported on the relationship between birthweight, taken as a marker of prenatal environment, and breast cancer, but with differing results. One study (Ekbom et al, 1992; 1997) reported no association between birthweight and breast cancer, two (Le Marchand et al, 1988; Sanderson et al, 1996) an inverse relationship, and one (Michels et al, 1996) a positive association. The latter was a case-control study nested within the US Nurses' Cohort, which found that the odds of breast cancer for women who weighed 4 kg or more at birth was twice that of women who weighed less than 2.5 kg (Michels et al, 1996). The relationship was strongest for women aged 50 or younger.

Various factors might explain the inconsistency of these results. In some studies the information on birthweight was based on recall by the women themselves or their mothers (Michels et al, 1996; Sanderson et al, 1996), in most no account was taken of adult-life risk factors for breast cancer (Ekbom et al, 1992; 1997; Le Marchand et al, 1988), and no account was taken in any of childhood and pubertal factors.

The present study examines the relationship between birthweight and breast cancer in a UK national cohort of 2221 women who have been followed since their birth in 1946, and for whom data on birthweight, markers of childhood growth and adult-life risk factors for breast cancer have been recorded.

*Received 10 March 2000*
*Revised 1 June 2000*
*Accepted 2 June 2000*

*Correspondence to*: BL De Stavola

### DATA AND METHODS

The Medical Research Council National Survey of Health and Development (NSHD) is a socially stratified birth cohort of 2548 women and 2814 men born in the UK during the week 3–9 March 1946 (Wadsworth, 1991; Wadsworth and Kuh, 1997). The cohort comprised single, legitimate births to wives of all non-manual and agricultural workers and to 1 in 4 wives of manual workers. There have been 19 follow-up contacts with the cohort members between their birth and age 43 years, most by home interviews. The sample interviewed at age 43 years were, in most respects, representative of the native population of that age (Wadsworth et al, 1992). Since 1993, when the cohort members were 48 years of age, a postal health questionnaire has been sent annually to all women in the study with whom there was still direct contact. At these separate contacts, breast cancer diagnosis was self-reported and recorded. In 1971, when the National Health Service Central Register (NHSCR) started to record cancers occurring in the population of the UK, all cohort members (including those with whom there was no longer direct contact) were 'flagged' at the NHSCR. This provided notification of registrations of cancer, death and emigration for the cohort.

Information on birthweight was extracted from the birth records of the cohort members and categorized into four groups: < 3.000 kg, 3.000–3.499 kg, 3.500–3.999 kg, ≥ 4.000 kg, in accord with previous studies (Ekbom et al, 1997; Michels et al, 1996). Data on maternal age and birth order were collected at the original home visit. Data on childhood and adult risk factors, that might confound or modify the relation of birthweight with breast cancer, were recorded at follow-up contacts. Childhood social class was assigned from father's occupation when the cohort member was aged 4 years or, if this was unknown, when aged 7 or 11. Height and weight were measured prospectively throughout childhood and adult life using standardized procedures (Stark et al, 1981); the values recorded at age 7 and 36 years were a priori selected for this analysis as markers of pre-pubertal growth and adult size, respectively. Age at menarche was obtained from questions to mothers when survey members were aged 11 and 15 years (Cooper et al, 1996) or, failing this, from the women themselves at the age of 48 years ($n$ = 101). The dates of all live births to the women in the cohort have been updated through follow-up contacts and were used to obtain age-at-first-live-birth and parity.

Of the 2548 women who had been in the birth cohort, 2221 (87%) were included in the analyses. Of the 327 who were excluded, 299 had died or emigrated before 1971, 17 could not be flagged and 11 had no birthweight recorded.

Follow-up was analysed from 1 January 1971, when the women were aged 24 years and 9 months. For those who had not returned any of the postal questionnaires ($n$ = 660), follow up was analysed until the earliest of the following: breast cancer diagnosis, death, emigration or 31 December 1992 (the last date for which the NHSCR cancer registration data were considered to be complete at the time of this analysis). For women who had returned at least one questionnaire ($n$ = 1561), follow-up was until the earliest of: breast cancer diagnosis or last completed questionnaire (up to 1997).

Since breast cancer may have a different aetiology at pre- and postmenopausal ages, events and follow-up prior to menopause were examined separately. Menopausal status could only be ascertained through the postal questionnaires, hence only women who responded to at least one questionnaire were considered. Two of these 1561 women gave insufficient information to determine menopausal status and were excluded. The premenopausal follow-up of the remaining 1559 women was then defined from 1 January 1971 to the earliest of: breast cancer diagnosis, date of natural menopause (defined retrospectively after 12 months of amenorrhoea), date of hysterectomy (or bilateral oophorectomy), start of hormone replacement therapy (HRT), or date of last completed questionnaire (up to 1997).

### Statistical methods

A Cox regression model was used in the analyses, with age as the time-scale of interest because of the expected associations between attained age, breast cancer incidence and most of the factors to be studied (Clayton and Hills, 1993). Plots of cumulative breast cancer incidence rates for separate birthweight categories were examined to assess their effect over different ages and to check the proportional hazards assumption underlying the Cox model. Univariate analyses provided age-adjusted rate ratios (RRs) by birthweight category. These were re-examined after adjustments for the effects of other perinatal and later-life factors. Since most of these potential confounders had considerable numbers of missing observations, their influences on the estimated effect of birthweight were examined separately. Subsets of women with information on each potential confounder were created and the age-adjusted RR for binary birth weight (≥ 3.5 kg vs < 3.5 kg) was compared with that additionally adjusted for the relevant factor. Potential confounding was judged on the extent of the relative difference between these two estimates. Effect modification was assessed via likelihood ratio tests for the significance of the interaction between birthweight and each categorical factor. Lastly, all these analyses were repeated with restriction to premenopausal ages.

### RESULTS

A total of 37 breast cancer cases occurred during the follow-up period. Twenty were identified through cancer registrations of which 14 (70%) were also self-reported and 17 were only self-reported. The median age at breast cancer incidence for the 37

**Table 1** Breast cancer incidence rates and age-adjusted rate ratios (RRs) for birthweight, by age

| Birthweight (kg) | Women *n* | (%) | Breast cancer cases (*n*) | Rate (per 100 000 p-yrs) | | Age-adjusted analysis RR | (95% CI) |
|---|---|---|---|---|---|---|---|
| **All ages** ($n$ = 2221) | | | | | | | |
| *Categorical* | | | | | | | |
| < 3.000 | 570 | (26) | 7 | 50.7 | | 1 | |
| 3.000–3.499 | 828 | (37) | 11 | 54.6 | | 1.05 | (0.41, 2.71) |
| 3.500–3.999 | 657 | (30) | 15 | 92.9 | | 1.76 | (0.72, 4.33) |
| ≥ 4.000 | 166 | (7) | 4 | 99.8 | | 2.02 | (0.59, 6.90) |
| | | | | | Test for linear trend[a] | $\chi^2_{(1)}$ = 2.25, $P$ = 0.13 | |
| *Binary* | | | | | | | |
| < 3.500 | 1398 | (63) | 18 | 53.0 | | 1 | |
| ≥ 3.500 | 823 | (37) | 19 | 94.3 | | 1.76 | (0.92, 3.35) |
| | | | | | Test for heterogeneity[b] | $\chi^2_{(1)}$ = 2.91, $P$ = 0.09 | |
| **Premenopausal ages**[c] ($n$ = 1559) | | | | | | | |
| *Categorical* | | | | | | | |
| < 3.000 | 385 | (24) | 2 | 22.7 | | 1 | |
| 3.000–3.499 | 588 | (38) | 6 | 45.1 | | 1.99 | (0.40, 9.86) |
| 3.500–3.999 | 479 | (31) | 8 | 73.4 | | 3.26 | (0.69, 15.36) |
| ≥ 4.000 | 107 | (7) | 3 | 124.6 | | 5.65 | (0.95, 33.84) |
| | | | | | Test for linear trend[a] | $\chi^2_{(1)}$ = 4.62, $P$ = 0.03 | |
| *Binary* | | | | | | | |
| < 3.500 | 973 | (62) | 8 | 36.2 | | 1 | |
| ≥ 3.500 | 586 | (38) | 11 | 82.7 | | 2.31 | (0.93, 5.74) |
| | | | | | Test for heterogeneity[b] | $\chi^2_{(1)}$ = 3.30, $P$ = 0.07 | |

[a]Likelihood ratio test calculated from the Cox regression model using the median value in each category as score; [b]Likelihood ratio test calculated from the Cox regression model for the significance of the ≥ 3.500 kg birthweight indicator; [c]The number of women included in these analyses is smaller than that used in the all-age analyses because menopausal status could only be ascertained for women who replied to at least one postal questionnaire

*© 2000 Cancer Research Campaign* 

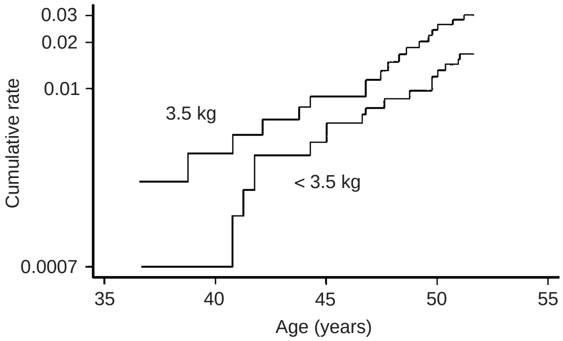

**Figure 1** Cumulative breast cancer incidence rates by birthweight cases was 46.8 years (range: 36.3–51.2) and the median age at the end of follow-up for the remaining 2184 women was 51.6 years (range: 25.2–51.6). Of the 1559 women for whom menopausal status was known at the end of follow-up, 19 were premenopausal breast cancer cases.

All-ages breast cancer incidence rates increased with increasing birthweight (Table 1). When the cumulative incidence rates for the four birthweight categories were plotted against attained age (on a logarithmic scale), they were roughly parallel, indicating that the birthweight effect on breast cancer risk was constant over time. Hence the age-adjusted RRs estimated by the Cox regression model represent the overall effects of birthweight over the entire age span analysed in this study. The RRs show a two-fold increase in breast cancer rates for women whose birthweight was at least 4 kg, relative to those who weighed less than 3 kg, and a consistent trend through the four weight categories (Table 1). Using a binary classification (< 3.5 kg vs ≥ 3.5 kg) the constant effect of birthweight, with consistently higher rates in the heavier birthweight group, was still evident (Figure 1). Breast cancer rates for women whose birthweight was greater than or equal to 3.5 kg were over 70% greater in comparison with those for women who were lighter (RR = 1.76, 95% CI: 0.92, 3.35; Table 1). The premenopausal analyses showed a stronger effect of birthweight with a steady increase in rates across the birthweight categories (test for linear

**Table 2** Rate ratios (RRs) for birthweight[a] with and without adjustment for selected potential confounders; all ages

| Potential confounder[b] | Women[b–c] (*n*) | Breast cancer cases[c] (*n*) | Age-adjusted analysis RR | Age-adjusted analysis (95% CI) | Age and confounder adjusted analysis RR | Age and confounder adjusted analysis (95% CI) | Test for interaction[d] |
|---|---|---|---|---|---|---|---|
| None | 2221 | 37 | 1.76 | (0.92, 3.35) | – | | – |
| Maternal age | 1929 | 31 | 1.98 | (0.97, 4.01) | 2.08 | (1.02, 4.23) | 0.09 |
| Birth order | 2220 | 37 | 1.76 | (0.92, 3.34) | 1.97 | (1.03, 3.78) | 0.29 |
| Childhood social class | 2101 | 34 | 2.09 | (1.06, 4.12) | 2.09 | (1.06, 4.12) | 0.55 |
| Weight at 7 years | 1850 | 31 | 2.02 | (0.99, 4.09) | 2.07 | (1.00, 4.29) | 0.23 |
| Height at 7 years | 1909 | 32 | 2.11 | (1.05, 4.24) | 2.00 | (0.98, 4.06) | 0.03 |
| Age at menarche | 1687 | 30 | 2.18 | (1.06, 4.49) | 2.21 | (1.07, 4.55) | 0.05 |
| Adult height | 1631 | 31 | 2.07 | (1.02, 4.19) | 1.95 | (0.95, 4.03) | 0.52 |
| Age at first birth | 1608 | 28 | 2.23 | (1.06, 4.72) | 2.24 | (1.06, 4.74) | 0.26 |
| Parity | 1608 | 28 | 2.23 | (1.06, 4.72) | 2.17 | (1.03, 4.60) | 0.21 |
| Weight at age 36 years | 1634 | 31 | 2.06 | (1.02, 4.18) | 2.03 | (0.99, 4.14) | 0.21 |

[a]Birth weight was included in the Cox regression model as a binary indicator coded <3.5 kg and ≥3.5 kg; [b]'maternal age' was coded as < 30, ≥ 30 years; 'birth order' as 1st, 2nd, 3rd, 4th or more; 'age at first birth' as < 20, 20–29, ≥ 30 years, nulliparous; 'parity' as 0, 1, 2, ≥ 3; 'childhood social class' as I & II, III non-manual, III manual, IV & V; all weight and height measurements were categorized into quartiles; 'age at menarche' was classified as < 12, 12–13, ≥ 14 years; [c]The number of women, and breast cancer cases, included in each analysis varied because of missing data for the different potential confounders; [d]*P*-value from likelihood ratio test for interaction between binary birthweight and the factor listed in each row

**Table 3** Breast cancer incidence rate ratios (RRs) in relation to birthweight and height at age 7 years, by age

| | Exposure categories: Birthweight (kg) | Exposure categories: Height at age 7[a] (m) | Women (*n*) | Breast cancer cases (*n*) | Age-adjusted analysis RR | Age-adjusted analysis (95% CI) |
|---|---|---|---|---|---|---|
| All ages (*n* = 1909)[b] | | | | | | |
| | < 3.5 | < 1.22 or ≥ 1.22 | 1194 | 14 | 1.00 | |
| | ≥ 3.5 | < 1.22 | 486 | 7 | 1.21 | (0.49, 2.99) |
| | ≥ 3.5 | ≥ 1.22 | 229 | 11 | 4.01 | (1.82, 8.83) |
| | | | | | Test for heterogeneity[d] | $\chi^2_{(2)} = 10.75$, $P = 0.005$ |
| Premenopausal ages (*n* = 1351)[c] | | | | | | |
| | < 3.5 | < 1.22 or ≥ 1.22 | 836 | 6 | 1.00 | |
| | ≥ 3.5 | < 1.22 | 345 | 3 | 1.23 | (0.31, 4.91) |
| | ≥ 3.5 | ≥ 1.22 | 170 | 7 | 5.86 | (1.97, 17.44) |
| | | | | | Test for heterogeneity[d] | $\chi^2_{(2)} = 9.77$, $P = 0.008$ |

[a]Categories based on quartiles of the height distribution: bottom three quartiles (< 1.22 m) vs top quartile (≥ 1.22); [b]The number of women included in this analysis is less than 2221 because of missing values for height at age 7; [c]The number of women included in this analysis is less than 1559 because of missing values for height at age 7; [d]Likelihood ratio test calculated from the Cox regression model for the significance of the heterogeneity of the RRs for the three exposure categories

 *© 2000 Cancer Research Campaign*

trend: $P = 0.03$); the RR corresponding to the binary classification of birthweight was greater than 2 (Table 1).

Table 2 shows the results of adjusting the all-ages RR for binary birthweight for different potential confounders. There is some evidence of confounding due to maternal age, birth order and adult height, as the age-adjusted RR for birthweight changes by at least 5% when additionally adjusted for any of these factors. A significant interaction between birthweight and quartiles of height at age 7 years ($P = 0.03$) is observed, with the effect of birthweight being stronger in taller girls. There is also a borderline significant interaction between birthweight and age at menarche ($P = 0.05$), with a stronger effect of birthweight in girls with earlier puberty. Adjustment for the other variables in the table as well as body mass index at ages 7 and 36 years (results not shown), did not affect the original birthweight estimates.

Since height at age 7 and age at menarche were negatively correlated (Spearman's correlation coefficient = –0.17, $P < 0.001$) the separate interactions between these two factors and birthweight are likely to be a reflection of the same process. Indeed the median age at menarche of the women in the top quartile of height at age 7 was 7 months earlier than that of women who were not so tall at 7 years. When the two interactions were included in the same model, only the one between birthweight and height at age 7 remained significant ($P = 0.04$), while that with age at menarche became redundant ($P = 0.18$).

To examine this further without creating too many strata, we regrouped height at age 7 as a binary variable (the first three quartiles vs the fourth, i.e. < 1.22 m vs ≥ 1.22 m) and modelled its interaction with birthweight using three groups: birthweight <3.5 kg (baseline); birthweight ≥ 3.5 kg and height at age 7 < 1.22 m; birthweight ≥ 3.5 kg and height at age 7 ≥ 1.22 m (Table 3). The baseline group included all low-birthweight girls without distinction by height at age 7 because of small numbers. The effect of this three-category factor on breast cancer rates was highly significant ($P = 0.005$). Women who were heavy at birth and short or average at age 7 were estimated to have a 21% increase in breast cancer rates, relative to women who were light at birth (RR = 1.21, 95% CI: 0.49–2.99). Women who were heavy at birth and tall at age 7 had a four-fold increase (RR = 4.01, 95% CI: 1.82–8.83). These results did not materially change after adjusting separately for each of the potential confounders considered in Table 2. There was evidence of a similar, although weaker, modification of the effect of birthweight due to height at age 6 ($P = 0.05$) and 11 ($P = 0.06$) but no modification due to height at ages 2 ($P = 0.34$) and 15 ($P = 0.19$).

In analyses repeated on data restricted to premenopausal ages, the interaction between birthweight and height at age 7 was slightly stronger than that for the complete data set, with those who were heavy at birth and tall at 7 years having an almost six-fold increase in breast cancer rates relative to those who were light at birth (Table 3).

### DISCUSSION

In a UK national cohort of women born immediately after World War II, birthweight was positively associated with breast cancer, which persisted after adjustment for other perinatal and later-life factors.

In agreement with previous studies (Michels et al, 1996; Sanderson et al, 1996) the effect of birthweight on breast cancer risk was particularly marked at premenopausal ages. Women who weighed 4 kg or more at birth were nearly six times more likely to develop breast cancer prior to menopause than those who weighed less than 3 kg. In contrast to the US Nurses' Cohort study (Michels et al, 1996), however, the effect of birthweight found in our study was not restricted to the extreme values of its distribution. Breast cancer risk at premenopausal ages rose gradually for successive categories of increasing birthweight, although the estimates were based on small numbers.

Our study had several strengths. Information on birthweight was obtained prospectively from routinely completed birth records and therefore was not affected by recall bias; data on birthweight were not available for only 0.5% of women. Incomplete follow-up was minimized because, as well as having frequent contacts, the cohort has been flagged through the NHSCR since 1971, losses before then being essentially due to deaths and emigrations at young ages. Of the 37 breast cancer cases identified, 14 were self-reported as well as identified through flagging, 16 were only self-reported because they occurred after 1992 (the last year for which NHSCR data are available) and six, although identified only through flagging, were women with whom there was no direct contact. Only one woman reported a diagnosis of breast cancer before 1992 but did not appear in the NHSCR system. The number of identified breast cancer cases appears therefore to be complete and indeed was similar to that expected on the basis of national incidence rates (37 observed, 36.8 expected). A final point in support of our results is that we were able to examine whether the effect of birthweight on breast cancer was confounded or modified by markers of childhood growth and adult-life risk factors for breast cancer.

The study also presented some limitations. First, as in some previous studies (Michels et al, 1996; Sanderson et al, 1996), there was no information on gestational age and it is conceivable that weight relative to gestational age may be more important than just birthweight. However, this would have led, if anything, to an underestimation of the effect of the growth process in utero on breast cancer risk. Second, the cohort is still relatively young and the number of breast cancer cases relatively small. Future re-analysis of follow-up data from this cohort, when more cases will have been accrued, will provide more precise estimates and allow separate analyses at postmenopausal ages. Last, information on potential confounding or modifying factors was not available for a substantial minority of the cohort members. However, the results obtained from the subsets of women with information on the different confounders were in broad agreement with each other, while those for women with data on height at age 7 were based on 86% both of the cohort (1909 out of 2221) and of the cases (32 out of 37). Hence it seems unlikely that the exclusions would explain the height results.

Although the effect of birthweight on breast cancer risk is compatible with the hypothesis that growth processes in utero are important in the aetiology of this tumour, our data suggest that exposures in early childhood are also relevant. Our study identified a strong statistical interaction between the effects of birthweight and height at age 7 (and, to a lesser extent, height at age 6 and 11, and age at menarche). The lack of interaction at age 2 argues against the interpretation that information on birthweight plus height at age 7 simply gives a better marker of fetal growth than information on birthweight alone. There was no significant interaction with height at 15 or adult height, suggesting that rate of childhood growth, rather than height per se, is the important factor. The results are therefore consistent with the hypothesis that nutri-

*© 2000 Cancer Research Campaign* 

tion during childhood may modulate the effect of birthweight on breast cancer risk (De Waard and Trichopoulos, 1988) since both height and age at menarche seem to be influenced by nutritional status early in life (Frisch, 1987; Stoll et al, 1994). A recent prospective study showing a strong association between leg length measured before age 8 years and breast cancer mortality lends support to this idea (Gunnell et al, 1998).

Birthweight has been found to be positively associated with high levels of pregnancy oestrogens in some studies (Petridou et al, 1990; Trichopoulos, 1990) and breast cancer with being a dizygotic twin (Braun et al, 1995; Swerdlow et al, 1997), which evidence suggests may be of hormonal origins (Nylander, 1981; Martin et al, 1984; Kappel et al, 1985). Moreover, the increase in breast cancer risk in dizygotic twins has been restricted to young ages, like the relationship with birthweight which we, and others (Michels et al, 1996; Sanderson et al, 1996), have found. High birthweight may also reflect the role of other hormones such as insulin-like growth factors (IGFs) which are important regulators of somatic growth during fetal life and childhood (Holly, 1998). High levels of IGFs may also result in an increased number of stem cells and/or increased mitosis in the mammary gland. A recent prospective study showed a strong association between adult circulating levels of IGF-I and later development of premenopausal (but not postmenopausal) breast cancer (Hankinson et al, 1998). So endocrine factors may underlie the processes that determine both size at birth and height at age 7, as well as their interaction.

In summary, our findings are consistent with the hypothesis that the effect of birthweight on breast cancer risk is subsequently modulated by childhood growth. They also emphasize the need to examine breast cancer aetiology by taking into account risk factors that operate pre-natally or at other stages of life (Kuh and Ben-Shlomo, 1997).

### ACKNOWLEDGEMENTS

We thank the Medical Research Council for funding this project and all the women who have participated, and still are participating, in the MRC Survey of Health and Development, for their patience and dedication.

### REFERENCES

Braun MM, Ahlbom A, Floderus B, Brinton LA and Hoover RN (1995) Effect of twinship on incidence of cancer of the testis, breast, and other sites (Sweden). *Cancer Causes Control* **6**: 519–524

Clayton D and Hills M (1993) *Statistical Models in Epidemiology*. Oxford University Press: Oxford

Cooper C, Kuh D, Egger P, Wadsworth M and Barker D (1996) Childhood growth and age at menarche. *Br J Obs Gyn* **103**: 814–817

De Waard F and Trichopoulos D (1988) A unifying concept of the aetiology of breast cancer. *Int J Cancer* **41**: 666–669

Ekbom A, Trichopoulos D, Adami H-O, Hsieh C-C and Lan S-J (1992) Evidence of prenatal influences on breast cancer risk. *Lancet* **340**: 1015–1018

Ekbom A, Hsieh C-C, Lipworth L, Adami H-O and Trichopoulos D (1997) Intrauterine environment and breast cancer risk in women: a population-based study. *J Natl Cancer Inst* **89**: 71–76

Frisch RE (1987) Body fat, menarche, fitness and fertility *Hum Reprod* **2**: 521–533

Gunnell DJ, Davey Smith G, Frankel S, Nanchalan K, Braddon FEM, Pemberton J and Peters TJ (1998) Childhood leg length and adult mortality: follow-up of the Carnagie (Boyd Orr) Survey of diet and health in pre-war Britain. *J Epidemiol Community Health* **52**: 142–152

Hankinson SE, Willett WC, Colditz GA, Hunter DJ, Michaud DS, Deroo B, Rosner B, Speizer FE and Pollak M (1998) Circulating concentrations of insulin-like growth factor-I and risk of breast cancer. *Lancet* **351**: 1393–1396

Holly J (1998) Insulin-like growth factor-I and new opportunities for cancer prevention. *Lancet* **351**: 1373–1375

Kappel B, Hansen K, Moller J and Faaborg-Anderson J (1985) Human placental lactogen and dU-estrogen levels in normal twin pregnancies. *Acta Genet Med Gemellol* **34**: 59–65

Kuh D and Ben-Shlomo Y (1997) *A life course approach to chronic disease epidemiology: tracing the origins of ill-health from early to later life*. Oxford University Press: Oxford

Le Marchand L, Kolonel LN, Myers BC and Mi M-P (1988) Birth characteristics of premenopausal women with breast cancer. *Br J Cancer* **57**: 437–439

Martin NG, El Beaini JL, Olsen ME, Bhatnagar AS and Macourt D (1984) Gonadotrophin levels in mothers who have two sets of DZ twins. *Acta Genet Med Gemellol* **33**: 131–139

Michels KB, Trichopoulos D, Robins JM, Rosner BA, Manson JE, Hunter DJ, Colditz GA, Hankinson SE, Speizer FE and Willett WC (1996) Birthweight as a risk factor for breast cancer. *Lancet* **348**: 1542–1546

Nylander PPS (1981) The factors that influence twinning rates. *Acta Genet Med Gemellol* **30**: 189–202

Petridou E, Panagiotopoulou K, Katsouyanni K, Spanos E and Trichopoulos D (1990) Tobacco smoking, pregnancy oestrogens and birth weight. *Epidemiology* **1**: 247–250

Sanderson M, Williams MA, Malone KE, Stanford JL, Emanuel I, White E and Daling JR (1996) Perinatal factors and risk of breast cancer. *Epidemiology* **7**: 34–37

Stark O, Atkins E, Wolff OH and Douglas JWB (1981) A longitudinal study of obesity in the National Survey of Health and Development. *BMJ* **283**: 13–17

Stoll BA, Vatten LJ and Kvinnsland S (1994) Does physical maturity influence breast cancer risk? *Acta Oncol* **33**: 171–176

Swerdlow AJ, De Stavola BL, Swanwick MA and Maconochie NES (1997) Risks of breast and testicular cancers in young adult twins in England and Wales: evidence on prenatal and genetic aetiology. *Lancet* **350**: 1723–1728

Trichopoulos D (1990) Hypothesis: does breast cancer originate in utero? *Lancet* **335**: 939–940

Wadsworth MEJ (1991) *The Imprint of Time: Childhood, History and Adult Life*. Oxford University Press: Oxford

Wadsworth MEJ, Mann SL, Rodgers B, Kuh DJL, Hilder WS and Yusuf EJ (1992) Loss and representativeness in a 43 year follow-up of a national birth cohort. *J Epidemiol Community Health* **46**: 300–304

Wadsworth MEJ and Kuh DJL (1997) Childhood influences on adult health: a review of recent work in the British 1946 national cohort study, the MRC National Survey of Health and Development. *Paediat and Perianat Epidemiol* **11**: 2–20

 *© 2000 Cancer Research Campaign*
